# Intracranial Extension of Pott’s Puffy Tumor in a Child: CT and MRI Findings

**DOI:** 10.1590/0037-8682-0249-2026

**Published:** 2026-10-16

**Authors:** Elif Gozgec, Seyda Guven

**Affiliations:** 1 Ataturk University School of Medicine, Department of Radiology, Erzurum, Turkey.

A 9-year-old boy presented with headache, rhinorrhea, and progressive forehead swelling. The patient had no history of trauma or underlying disease. Physical examination revealed a firm, approximately 3 cm in the left frontal swelling. Laboratory tests showed 10.9 ×10⁹/L white blood cell count, 80 mm/h erythrocyte sedimentation rate, and 129 mg/L C-reactive protein level. Contrast-enhanced brain computed tomography (CT) showed increased attenuation in the frontal and ethmoid sinuses. A 33 × 12 × 11 mm peripherally enhancing hypodense collection was identified adjacent to the anterior wall of the frontal bone, consistent with a subperiosteal abscess. Cortical irregularities and erosive changes indicated frontal bone osteomyelitis. A 71 × 45 × 20 mm epidural abscess was observed (Figure 1). Contrast-enhanced magnetic resonance imaging (MRI) demonstrated continuity between the epidural abscess and subperiosteal collection, with adjacent leptomeningeal enhancement (Figure 2). The patient underwent surgical drainage. Cultures of the purulent material yielded Gram-positive anaerobic cocci. The patient received intravenous antibiotics and was discharged without neurological sequelae. Pott’s puffy tumor is a rare clinical entity characterized by a osteomyelitis-associated subperiosteal abscess. It often occurs in teenagers and adolescents. The infection may extend inward, leading to serious intracranial complications. CT delineates osseous involvement, whereas MRI more accurately identifies intracranial extension^1^ ^,^ ^2^.Early diagnosis and prompt multidisciplinary management are essential to prevent potentially life-threatening complications^1^ ^,^ ^3^. 

**FIGURE 1:**
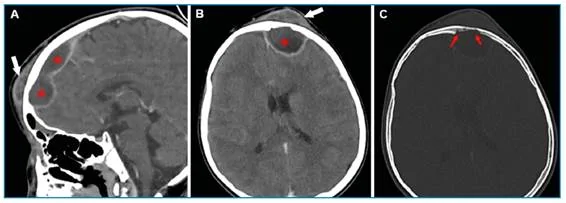
Contrast-enhanced CT images demonstrate a frontal epidural abscess and associated complications. **(A,B)** On saggital and axial images, a peripherally enhancing epidural abscess (asterisks) is observed adjacent to the frontal bone, with an overlying subperiosteal empyema (arrows). **(C)** The bone window image shows cortical irregularity and erosion of the frontal bone (arrows), consistent with osteomyelitis.

**FIGURE 2:**
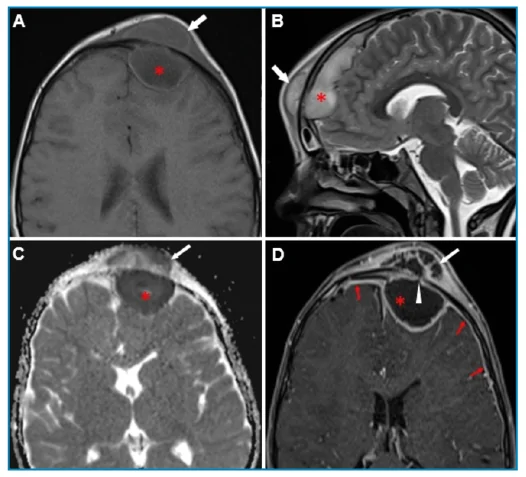
MRI demonstrates the epidural abscess and its extension. **(A)** Axial contrast-enhanced T1-weighted image reveals a peripherally enhancing epidural abscess (asterisk), with an overlying subperiosteal collection (arrow). **(B)** Sagittal T2-weighted image shows the epidural collection (asterisk) and associated soft-tissue component (arrow). **(C)** Diffusion-weighted imaging demonstrates restricted diffusion within the abscess (asterisk). **(D)** Axial contrast-enhanced T1-weighted image reveals continuity between the epidural abscess and subperiosteal collection, along with adjacent leptomeningeal enhancement (arrows).
